# A maturity model for Clinical Trials Management Ecosystem

**DOI:** 10.1017/cts.2024.1168

**Published:** 2025-01-22

**Authors:** Shruti Sehgal, E. Chris Pua, Svetlana Rojevsky, Michael J. Becich, Joshua Fehrmann, Boyd M. Knosp, Adam Wilcox, Jeffery C. Talbert, Catherine K. Craven, Justin Starren

**Affiliations:** 1 Northwestern University Feinberg School of Medicine, Chicago, IL, USA; 2 Vanderbilt Institute for Clinical and Translational Research, Vanderbilt University Medical Center, Nashville, TN, USA; 3 Tufts Clinical and Translational Science Institute, Tufts University, Boston, MA, USA; 4 Department of Biomedical Informatics, School of Medicine, University of Pittsburgh, PA, USA; 5 Clinical and Translational Science Institute, University of Minnesota, USA; 6 Roy, J. and Lucille A. Carver College of Medicine and the Institute for Clinical & Translational Science, University of Iowa, Iowa City, IA, USA.; 7 Institute for Informatics, Data Science and Biostatistics, Department of Medicine, Washington University in St Louis, St Louis, MO, USA; 8 Institute for Biomedical Informatics, University of Kentucky, Lexington, KY, USA; 9 University of Texas Health Science Center San Antonio, San Antonio, TX, USA; 10 University of Arizona, Tucson, AZ, USA

**Keywords:** Clinical Trials Management Ecosystem, clinical trials, maturity models, informatics, clinical and translational research

## Abstract

**Introduction:**

Managing clinical trials is a complex process requiring careful integration of human, technology, compliance, and operations for success. We collaborated with experts to develop a multi-axial Clinical Trials Management Ecosystem (CTME) maturity model (MM) to help institutions identify best practices for CTME capabilities.

**Methods:**

A working group of research informaticists was established. An online session on maturity models was hosted, followed by a review of the candidate domain axes and finalization of the axes. Next, maturity level attributes were defined for min/max levels (level 1 and level 5) for each axis of the CTME MM, followed by the intermediate levels. A REDCap survey comprising the model’s statements was then created, and a subset of working group members tested the model by completing it at their respective institutions. The finalized survey was distributed to all working group members.

**Results:**

We developed a CTME MM comprising five maturity levels across 11 axes: study management, regulatory and audit management, financial management, investigational product management, subject identification and recruitment, subject management, data, reporting analytics & dashboard, system integration and interfaces, staff training & personnel management, and organizational maturity and culture. Informaticists at 22 Clinical and Translational Science Award hubs and one other organization self-assessed their institutional CTME maturity. Respondents reported relatively high maturity for study management and investigational product management. The reporting analytics & dashboard axis was the least mature.

**Conclusion:**

The CTME MM provides a framework to research organizations to evaluate their current clinical trials management maturity across 11 axes and identify areas for future growth.

## Introduction

The management of modern-day clinical trials is an arduous process that is continuing to increase in complexity. There are many moving parts across a multidisciplinary ecosystem of stakeholders. Effective planning and implementation of a clinical trial greatly influences its success. The integrated trial management systems and electronic health records (EHRs), data interoperability, and best practices to support clinical and translational researchers play a crucial role in improving the quality and efficiency of clinical trial management and research outcomes. In particular, academic medical centers with the Clinical and Translational Science Award (CTSA) maintain an integrated research and training environment for clinical and translational sciences. CTSA hubs play a central role in their local environments where they coordinate and collaborate with multiple spokes such as affiliated hospitals, clinics, and community health centers.

Standardizing and optimizing the management of clinical trials is key to accelerating the clinical trials process, improving study quality, and ensuring the accuracy, reliability, and integrity of the results. While the adoption of clinical trial management systems (CTMSs) has advanced standardizing the work involved in managing clinical trials, CTMSs typically only address a subset of the activities involved in conducting a clinical trial. Organizational leadership needs to regularly evaluate both CTMS and non-CTMS clinical trial activities to effectively run clinical trials and take appropriate action in order to stay up to date with changes in the technology and processes that comprise the broader Clinical Trials Management Ecosystem (CTME). However, self-evaluation tools developed by the CTSA consortium only assess the extent to which an institution has installed a CTMS. No adaptable frameworks or guidance exist to evaluate and track broader organizational clinical trial development, function, or behavior.

Increasingly, leaders turn to maturity models to address such questions [1,2]. They use maturity models to assess their team’s/ organization’s performance in a particular domain, recognize where their current methods fall short, identify areas for improvement, set improvement goals, and monitor progress over time. Importantly, these models can guide institutional strategic investment decisions. Maturity models are instruments to define and facilitate organizational management with regard to a particular function or behavior. Maturity models have existed since at least the 1970s [3]. Initially, they described “Stages of Growth” for process assessment and improvement. Maturity models are “based on the premise that people, organizations, functional areas, processes, etc., evolve through a process of development and growth toward a more advanced maturity accomplishing several stages.” [1] They became more formalized with the development of the Capability Maturity Model (CMM) in the 1980s [4]. The CMM utilizes a 5-level template, ranging from Initial (unpredictable and poorly controlled) through Optimizing (focus on continuous quality improvement) [4]. Under the CMM approach, the institution evaluates descriptive statements associated with each of the levels and then chooses the level that *most closely* describes the organization, realizing that some statements associated with a given level may not be a perfect match. This template has been adopted by many other models. When Garcia-Mireles et al. performed a systematic literature review on maturity models in 2012 across the software engineering literature, over 1,500 maturity model publications were identified [5].

While maturity models have been developed for many heterogeneous domains, none exist for managing the components and complexity of technical, regulatory, financial, and organizational characteristics related to clinical trials. Recently, maturity models have received considerable interest in information technology (IT) for healthcare [6– 9] and research. Perhaps, the most well known is the HIMSS Electronic Medical Record Adoption Model (EMRAM) [10]. Many other maturity models have been developed, including the Healthcare Analytics Adoption Model [11], the Enterprise Data Warehouse for Research (EDW4R) model [12,13], the research data sharing maturity model [14], Research IT [15], and recently, the Social Determinants of Health Maturity Model [16].

Several of the above models were developed by the CTSA consortium [17]. Within individual CTSAs, maturity models are used to assess the quality and efficiency of various translational informatics capabilities and activities. Informatics leadership at each CTSA is able to determine the areas that need to be prioritized by analyzing their self-assessment results and benchmarking across CTSA hubs [16]. When aggregate values across sites are evaluated, the results can be used to identify areas for multi-hub collaboration to improve quality across the consortium.

This article details the development of a CTME maturity model that can help inform research informatics, IT, and clinical trials leadership within institutions in several ways. Key uses include understanding perspectives across the organization, identifying gaps, developing best practices for CTME capabilities at their institution, generating source documents for internal discussion and planning, outlining a roadmap for future growth within the CTME domain, ultimately advancing the field of translational informatics.

## Methods

**Figure 1.**
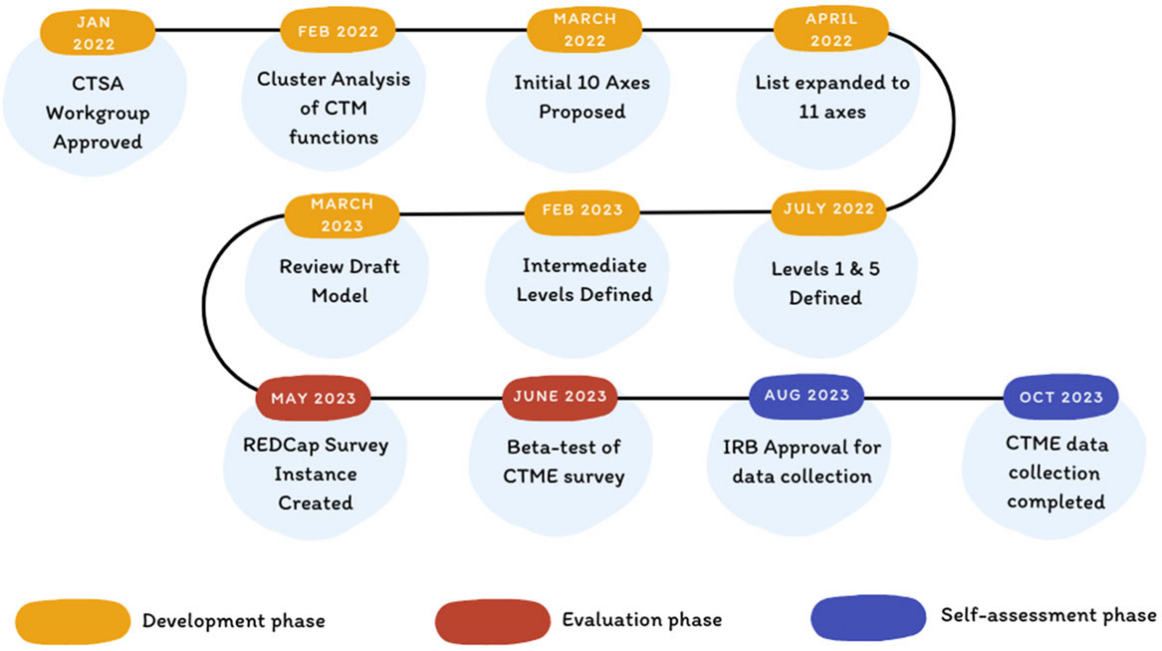
Process diagram illustrating the steps in the development of the Clinical Trials Management Ecosystem maturity model. CTSA = Clinical and Translational Science Award, CTM = clinical trials management, CTME = Clinical Trials Management Ecosystem, IRB = Institutional Review Board.

As detailed below, the development of the CTME maturity model was a multi-phase consensus-based process. In this paper, we describe the development of the CTME maturity model across three phases: development, evaluation, and self-assessment (Fig. 1).

### Development Phase

The first step toward developing a multi-axial CTME maturity model was to establish a working group of research informatics leaders from academic medical centers across the USA, especially those with CTSA or Institute for Clinical and Translational Research (ICTR) grant awards. Experts were identified through the CTSA Informatics Enterprise Committee and the American Medical Informatics Association Informatics (AMIA) Clinical Research Informatics Workgroup. A snowball sampling was employed, inviting those participating to suggest others whose experience was relevant. The working group ultimately consisted of 42 experts.

The candidate domain axes were identified based on an internal project at Northwestern University, led by Dr Firas Wehbe, which was aimed at developing a feature matrix for evaluating clinical trials management functions. The axes represented discrete parts of the clinical trials process. An online information session on maturity models was hosted for the working group members in March 2022. After an overview of maturity models, project goals, and expectations, we presented the candidate axes (n = 10) with experts from CTSA hubs. Based on working group recommendations, we modified one of the proposed axes, “Subject management” and divided it into “Subject identification and recruitment” and “Subject management” to provide granularity and specificity to the respective axes. As outlined in Table 1, 11 axes were finalized in April 2022 with expert input to construct the maturity model tool.

**Table 1.** Clinical Trials Management Ecosystem (CTME) maturity model axes and descriptions

Maturity model axis	Brief description
Study management	Protocol development, routing, and review, case report form development, contract management.
Regulatory and audit management	Electronic study binder, IRB submissions and approvals.
Financial management	Charge capture, budgeting, and pricing.
Investigational product management	Medical equipment, drugs, biomaterial management, research pharmacy, etc.
Subject identification and recruitment	Cohort identification, screening, eligibility, consenting, etc.
Subject management	Patient study calendar, quality control, adverse event and cohort management, patient ranking, screening, eligibility, etc.
Data	Includes data acquisition and management-21 CFR Part 11 compliance, data quality, standards-based data management and ontologies, data sharing, and governance.
Reporting analytics & dashboard	Report generation, visualization, and dashboards.
System integration and interfaces	eIRB, barcode system, enterprise resource planning.
Staff training & personnel management	Training resources for staff.
Organizational maturity and culture	Leadership commitment to be a leading clinical research organization, clinical trials office, or equivalent.

A generic description of maturity levels outlined in Table 2 served as a foundation for developing maturity statements for every axis. We used the standard five levels of the CMM, which is the most common in maturity model development [18,19]. Study data were collected and managed using Research Electronic Data Capture (REDCap) application hosted at Northwestern University. REDCap is a secure, web-based software platform designed to support data capture for research studies [20,21]. We developed REDCap surveys to solicit candidate statements for each maturity level of each axis. After candidate statements were collected, they were discussed and revised in monthly virtual working group meetings. Because of the need to extensively revise the precise wording of each maturity statement, the consensus process was not possible via online tools only. Each maturity statement was discussed by the working group and refined together until consensus was achieved. This process was first applied to maturity level statements for min/max levels (level 1 and level 5) for each axis of the CTME maturity model. Next, attributes for the intermediate levels, that is, levels 2–4, were collected sequentially via REDCap and iteratively refined for all 11 axes. The statements for every level provide a detailed explanation of the stages from the initial (*ad hoc*) stage to the optimizing (efficient) level. The goal of this maturity model was to offer institutions a structured means to identify their current level of maturity in clinical trials management, as well as to understand gaps to address in strategic planning.

**Table 2.** Generic descriptions of maturity levels for Clinical Trials Management Ecosystem (CTME) maturity model

**Level 5- Optimizing (Efficient)**This is the top level – as good as it is going to get. It is the idealized state. It is a characteristic of processes and systems at this level that the focus is on continually improving process performance through both incremental and innovative technological changes/improvements. At maturity level 5, processes are concerned with addressing statistical common causes of process variation and changing the process (e.g., to shift the mean of the process performance) to improve process performance.
**Level 4- Quantitatively managed (Capable)**It is characteristic of processes and systems at this level that using process metrics, effective achievement of the process objectives can be evidenced across a range of operational conditions. The suitability of the process in multiple environments has been tested and the process refined and adapted. Process users have experienced the process in multiple and varied conditions and are able to demonstrate competence. The process maturity enables adaptations to particular projects without measurable losses of quality or deviations from specifications. Process Capability is established from this level. (Example – surgeon performing an operation hundreds of times with levels of negative outcome approaching zero).
**Level 3- Defined (Aspiring)**It is characteristic of processes and systems at this level that there are sets of defined and documented standard processes established and subject to some degree of improvement over time. The processes may not have been systematically or repeatedly used – sufficient for the users to become competent or the process to be validated in a range of situations. This could be considered a developmental stage – with use in a wider range of conditions and user competence development the process can develop to the next level of maturity.
**Level 2- Localized (Developing)**It is characteristic of this level of maturity that some processes are repeatable, possibly with consistent results. Process discipline is unlikely to be rigorous, but where it exists, it may help to ensure that existing processes are maintained during times of stress.
**Level 1- Initial (*Ad hoc*)**Lowest possible level. This is where you are if you are just starting out. It is characteristic of processes at this level that they are (typically) undocumented, and in a state of dynamic change, tending to be driven in *ad hoc*, uncontrolled, and reactive manner by users or events. This provides a chaotic or unstable environment for the processes.

### Evaluation Phase

By February 2023, maturity level statements for all axes were defined and the first version of the model was reviewed with the workgroup members (virtually via Zoom and online via REDCap) during March–April 2023 to solicit comments about the maturity model. A REDCap survey for the model’s statements was then created, and the working group members were asked to test the model by completing the survey at their respective institutions. The survey asked respondents to choose the best fit when selecting levels, acknowledging that an organization may satisfy most, but not all, of the statements regarding the best match level. They were also asked to submit feedback about the instrument itself. At this stage, the model was tested by five working group members. No modifications to the model’s design and content were suggested by any of the experts at this stage.

### Self-Assessment Phase

This survey for collecting information about the level of institutional CTME maturity was distributed in August 2023 to all working group members. Twenty-three academic medical centers (four from the Northeast, four from the West, seven from the Midwest, four from the Southwest, and four from the South) completed the self-assessment for their institution between August 2023 and October 2023. Although this evaluation was coordinated by an informatician at each academic medical center, anecdotal reports from many sites indicated that the responses for specific axes were solicited from a variety of clinical trials professionals, including Institutional Review Boards, research finance, regulatory oversight, clinical research centers, research administration leadership, IT leaders, and CTMS management.

### Ethical Considerations

This project was determined to be exempt by Northwestern University Institutional Review Board (IRB# STU00219631).

## Results

A consensus-based approach was used to develop a multi-axial CTME maturity model comprising five advancing levels of maturity across 11 axes of study management, regulatory and audit management, financial management, investigational product management, subject identification and recruitment, subject management, data, reporting analytics & dashboard, system integration and interfaces, staff training & personnel management, and organizational maturity and culture. Maturity level statements were clustered around three conceptual components of standardization, complexity/integration, and monitoring, thus providing an informatics-centric perspective to every maturity level. For example, the first level of regulatory and audit management will typically be characterized by *ad hoc*, paper-based processes that lack a well-defined, well-documented, controlled, and standardized mechanism, with no established framework for measuring and monitoring performance or milestones. At level 2, some key processes are standardized. Event reporting systems such as adverse event and protocol deviation have been implemented, but response to adverse events is still largely manual. Performance metrics are measured for key areas only. At the next level, institution-wide standardized audit policies exist and there is an established electronic framework for measuring and monitoring performance. Advancement to level 4 would feature the use of standardized metrics for process management and control across the institution, consistent success activating studies on time and reaching study milestones, automatic monitoring of study milestones, though intervention on specific studies would often be *ad hoc*. At the top level, well-integrated systems exist that provide interactive and actionable information that can be easily accessed by the main institutional clinical research and trials office and audit reports are available for review, decision support, and follow-up.

For each level of each CTME axis, three to five statements were developed under each cluster, resulting in over 200 statements (See Supplementary material 1 for maturity statements for the 11 axes of CTME maturity model). Exemplar statements for the CTME maturity model axes have been outlined in Table 3.

**Table 3.** Clinical Trials Management Ecosystem (CTME) maturity model axes with exemplar statements

1. Study management
Level 5Electronic systems for study management processes are all implemented and centralized across the institution. (Clinical trial protocols, contracts, case report forms, Institutional Review Board (IRB) submissions etc.)Level 4Electronic systems for study management processes (clinical trial protocols, contracts, case report forms, IRB submissions, etc.) are implemented/integrated with minimal need for manual communication between systems (e.g., external IRB web application does not “read” protocols automatically, or EHR does not automatically flag patients on study).Level 3Major systems like the Clinical Trials Management System (CTMS) and IRB are connected, but not all CTM systems. Electronic study management systems exist for most processes, increasing potential for the full electronic integration.Clear path to integration of processes. (Clinical trial protocols, contracts, case report forms, IRB submissions etc.)Level 2Localized standardization: Electronic study management systems for many processes at the department or center level (e.g., within a Cancer Center), but little institutional standardization and not necessarily electronically integrated.Level 1*Ad hoc*, manual processes (clinical trial protocols, contracts, case report forms, IRB submissions, etc.) carried out by independent systemsLack of a well-defined, well-documented, controlled, and standardized mechanism
2. Regulatory and audit management
Level 5Well-integrated systems that provide interactive and actionable information that can be easily accessed by the main institutional clinical research and trials office.Level 4Some components of audit reports are automatically generated using data from CTMS but may still require double documentation.Level 3Regulatory binder procedures require all ICH E6 essential documents to be included and managed as controlled documents – that is, the regulatory binder procedures are complete (over Good Clinical Practice (GCP)), apply to all studies regardless of funding source or institutional unit/school/department managing the study.Level 2Event reporting systems such as adverse event (AE) and protocol deviation have been implemented, but response to AEs is still largely manual.Level 1Regulatory and audit paper forms processes.*Ad hoc* processes by regulatory and audit management staff and study teamsDifficulty activating studies on time and hitting timeline milestones.
3. Financial management
Level 5CTMS is tightly integrated with electronic health records (EHRs); automatic, protocol-driven assignment of research versus clinical charges.Level 4Full CTMS, EHR, and financial integration for high-priority charge capture, with a defined process for completing in other areas.The processes are auditable (done most efficiently and effectively using system data or other artifacts generated through the process itself).Level 3Institutional procedures cover the full life cycle of pricing, budgeting, financial reporting, and management of clinical studies and apply to all studies regardless of funding source or institutional unit/school/department managing the study.Level 2Clinical trial study budget pricing lives in an excel document emailed out to teams.Level 1No integration with hospital billing. The business office manually charges insurance or the grant.Lack of up-to-date, comprehensive charge master, resulting in inconsistent costing among studies.
4. Investigational product management
Level 5Investigational product use is charged automatically by an integrated system to the grant.Level 4Organization has implemented a formal controlled document procedure for investigational product management.Level 3Institution-wide policies and physical infrastructure for managing investigational products exist, but the implementation of those policies may be inconsistent among research groups.Level 2Many but not all institutional groups managing clinical studies have procedures and physical infrastructure for managing investigational products when the institution is a site in a multicenter study.Single site studies may or may not have formalized procedures.Level 1*Ad hoc*, paper-based tracking of stored equipment and drugs, checklist for management.No electronic system in place for managing investigational products.
5. Subject identification and recruitment
Level 5Integrated system for managing multiple recruitment strategies across varied populations.Clinical research/trials management informs and improves EHR data collection (e.g. race, ethnicity, Social Determinants of Health, etc.).Level 4Standard examples of integrating identification with recruitment activities.Level 3Possible competition for recruitment between study teams.Level 2Recruitment coordination at the department or center level, but not institution-wide.Level 1Inconsistent communication to care providers about research opportunities.Patients may be contacted by multiple simultaneous studies.
6. Subject management
Level 5CTMS is the single source of truth for subject management.Centralized AE reporting, management, and adjudication systems.Level 4Institutional CTMS tracks subjects on all clinical trials.Institution has standardized on a central patient study calendar system.Level 3Institutional CTMS tracks subjects on interventional clinical trials, but not all studies.Level 2CTMS exists but not all features are enabled, and it is not yet used for all studies to track enrollment.Level 1*Ad hoc* systems for AE developed by individual investigators, resulting in delayed or missed AE reporting.
7. Data capture and CRF
Level 5Data models and standards are constantly updated and refined to support operational needs and align with national and international standards.Level 4Institutional procedures for management of clinical study data are standardized across the institution in alignment with national and international data standards, such as National Institutes of Health (NIH), Common Data Elements (CDEs), Clinical Data Interchange Standards Consortium (CDISC), and Observational Medical Outcomes Partnership (OMOP).Level 3Institution-wide policies for managing data exist, but the implementation of those policies may be inconsistent among research groups.Level 2Many but not all institutional groups managing clinical studies have procedures for managing data when the institution is a site in a multicenter study.Single-site studies may or may not have formalized data management procedures.Level 1No standardized solution in place.*Ad hoc* process by service providers, study teams, and requestors. Paper-based data collection for clinical trials.
8. Reporting, analytics, and dashboard
Level 5Reporting dashboards are available for all departments via tools like PowerBi or tableau, dynamically linked to electronic data warehouse (EDW) tables.Level 4Centralized, institutional resources exist for reporting and analytics.Highly effective business analytics beginning to impact clinical services esp. with respect to quality improvement.Level 3Institutional procedures for planned and *post hoc* analyses specify the documentation to be generated by the procedures to enable process control.Level 2Reports and dashboards provide information but are difficult to convert into process improvement projects.Level 1No dashboards available. Simple tables or spreadsheets.
9. System integration and interfaces
Level 5All systems are tightly integrated but loosely coupled via a Service Oriented Architecture (SOA).Standards-based interfaces. IHE (Integrating the healthcare enterprise), FHIR, and other electronic interfaces.Level 4Institutional policies for process and system integration and interfaces between systems are standardized across the institution.Level 3Initial work on integration of major systems (e.g., EHR, CTMS, IRB).Institutional focus on integration of clinical care systems, with relatively little institutional focus on integration between clinical and research systems.Level 2Systems may exist but are minimally integrated with other systems and use is not standardized and may be redundant.Level 1No integration among various systems, except *ad hoc* study-specific data integrations
10. Staff training and personnel management
Level 5Web-based, scalable trainings (GCP, HSP, or specific study trainings) available.Centralized tracking of relevant training with automated reminders.Level 4Centralized, institutional resources exist for staff training.Web-based systems to track and manage training requests.Level 3Institutional procedures for CR workforce training and management specify the documentation to be generated by the procedures to enable process control.Some groups have adopted centralized, online training resources.Level 2Systems may exist but are minimally integrated with other systems and use is not standardized and may be redundant.Level 1Training for staff exists but timing and needs are not always well matched.
11. Organizational maturity and culture
Level 5Top-level leadership commitment to be a leading clinical research organization.Centralized research (CR) organization is well thought out and structured to accomplish a well-defined set of missions and goalsLevel 4Top-level leadership across the organization is committed to achieving a set of clinical research missions and goals.Centralized CR governance group includes some, but not all, stakeholders.Level 3Institutional leadership acknowledges the importance of clinical research, but support throughout the organization is variable.CR governance is assigned to another institutional governance group, no dedicated CR governance group.Level 2Gaps in organizational structure are recognized and beginning to be addressed but no organizational priority to grow clinical research.Level 1Research organizational structure is outdated and/or poorly structured and does not align with workflow, function, and responsibilitiesGaps in organizational structure resulting in confusion, inconsistency, and redundancy.Frequent conflict/competition among multiple research groups.

### CTME Maturity Self-Assessment Results

Twenty-three academic medical centers representing 22 different CTSA hubs (out of 64 CTSA hubs) [22] and 1 non-CTSA academic medical center across the USA self-assessed their institutional maturity with regard to the CTME. Self-assessment representing a third of the country’s CTSA hubs, with no modifications proposed by any of the participating experts during the evaluation phase, suggests reasonable real-world applicability (see Supplementary material 2 for a summary of CTME maturity scores by institution).

**Figure 2.**
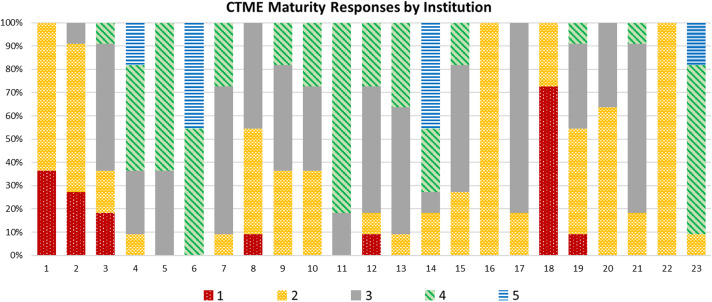
Institutional responses (n = 23*)* to the CTME maturity model self-assessment. CTME = Clinical Trials Management Ecosystem.

**Figure 3.**
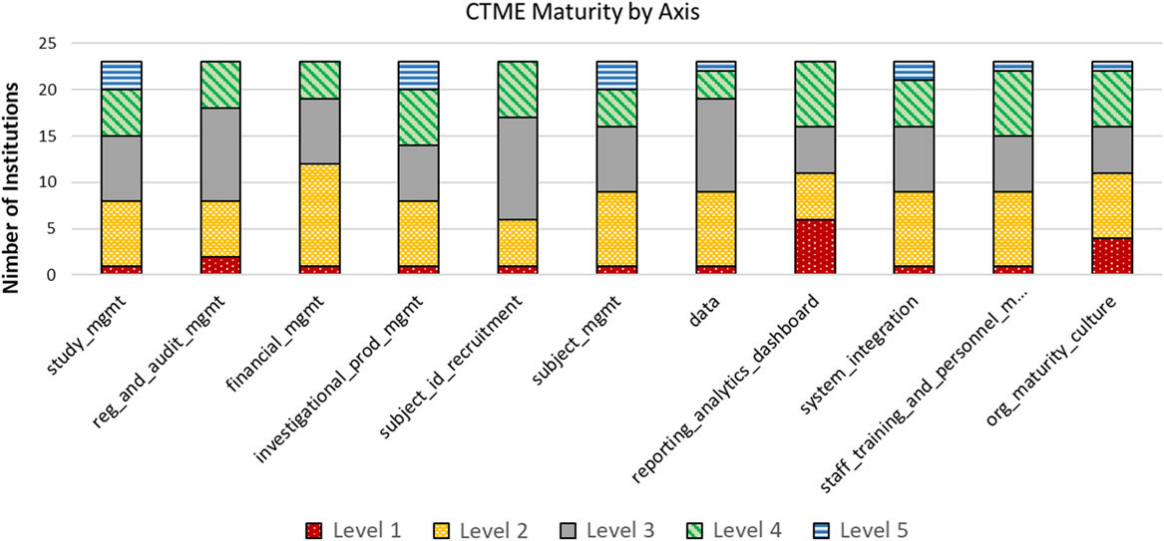
Figure depicting the maturity of institutions by axis with regard to the CTME. CTME = Clinical Trials Management Ecosystem.

Fig. 2 depicts the institutional responses to the CTME maturity model self-assessment. The average maturity across all axes and all institutions was 2.54 (SD: 0.50). With regard to the axes, the average ranged from 2.57 to 3.09. Fig. 3 shows the number of institutions endorsing a specific maturity level (i.e., self-assessment of the institution at that level) for every axis. The majority of institutions indicated a higher maturity for study management and investigational product management, while the reporting analytics & dashboard axis was the lowest scored axis. The CTME maturity across institutions ranged from 1.27 to 4.45.

## Discussion

The CTME maturity model defines five levels of evolutionary growth across 11 axes with the levels ranging from level 1, characterized by initial/*ad hoc* processes, to level 5, characterized by optimizing and efficient capabilities. This assessment can help determine the level of maturity of organizations of various sizes or units within a larger organization, thus demonstrating a balance in the applicability of the CTME maturity model. (The default definition of “institution” or “organization” here was an academic medical center that attempts to function as a cohesive entity regarding clinical research, even if that contains multiple corporate entities.) The CTSA consortium has developed a suite of maturity models to assist CTSA hubs in self-assessment and strategic planning [13– 17]. This model fills a gap in that suite by providing a more granular assessment of CTME.

Preliminary discussions of the working group were aimed to determine the range of challenges to improving clinical trials efficiency at the CTSA hubs. The most significant barriers that we identified included lack of IT infrastructure, lack of data systems or lack of integration of existing systems, existence of patient- and protocol-level data in siloed, fragmented systems, administrative and fiscal issues, recruitment issues, lack of institutional leadership commitment, and the availability of needed personnel and expertise [23,24]. All of these discussion findings informed the development of a formal framework for CTME. For example, the five advancing levels of maturity across system integration and interfaces data, financial management, subject identification and recruitment, subject management, staff training & personnel management, and organizational maturity and culture axes illustrate areas of intervention critical to advancing the respective domain and overcoming the barriers that have been described above. Further, several organizational benefits can be ascribed to the development and implementation of the CTME maturity model: 1) an improved understanding of the components and complexity of technical and organizational issues related to clinical trials management by the CTSA hubs [25]; 2) the establishment of consortium-wide benchmarking for CTME [15]; 3) addressing a missing component to the current suite of CTSA maturity models; 4) an improved ability of informatics, clinical trials, and senior institutional leadership to identify and remove the barriers to effective CTM at their respective hubs, provide insights to develop concrete plans, and empower informatics leads to advocate for CTM technology to institutional leadership. Some institutions are already using the CTME maturity model for internal strategic planning (personal communication, Starren and Knosp). Recently, the Iowa CTSA used the CTME maturity model to assess Iowa’s maturity as part of an interdisciplinary clinical trials strategic planning process. The team reported that the model was effective in identifying organizational gaps and improving clinical research operations [26]. The long-term impact will be improvement in the quality and comprehensiveness of CTME at CTSA hubs and other academic medical centers.

**Figure 4.**
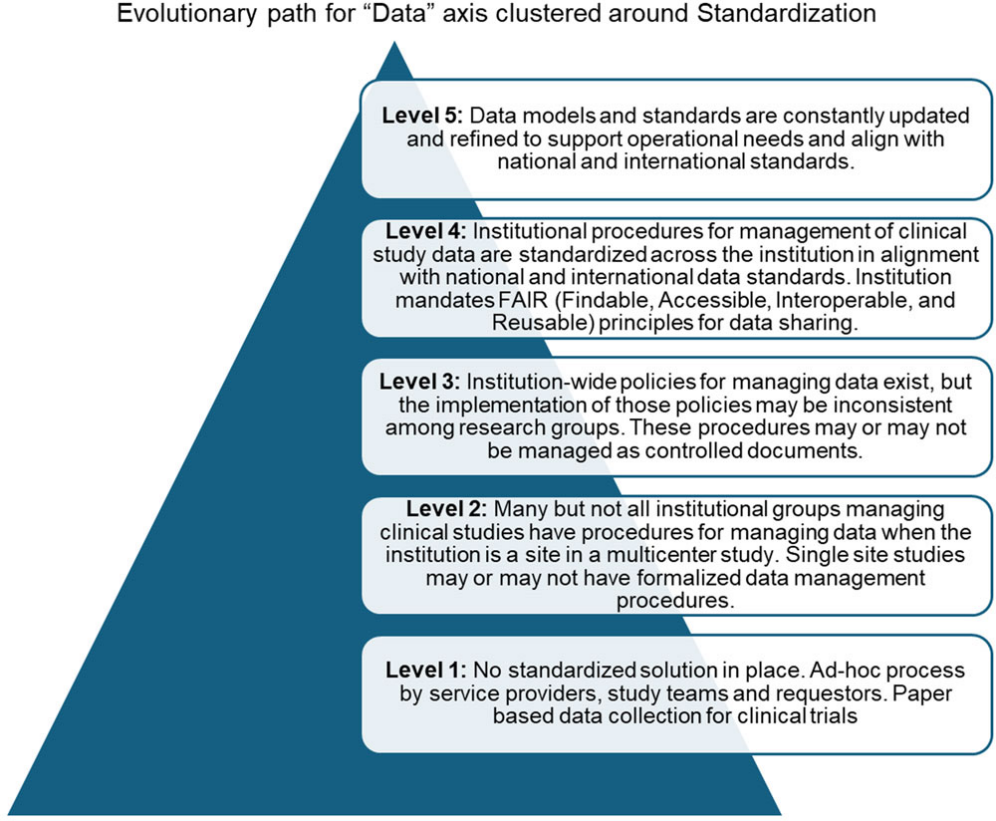
Figure illustrating the evolution of maturity for the “Data” axis of the CTME maturity model. *Note*: the illustration represents maturity statements clustered around one conceptual component only, standardization. The figure does not represent maturity statements for complexity/integration and monitoring. CTME = Clinical Trials Management Ecosystem.

Defining the evolutionary path consisted of first describing the characteristics of the initial and optimizing maturity stages or the lowest and highest levels (levels 1 and 5) for every CTME axis. This was followed by a description of anticipated, typical, logical, and desired evolution paths (Fig. 4). This top-bottom approach was important to ensure that the CTME MM was designed not only for descriptive purposes but also for wide application in practice, and it reflects the rigor of the intellectual process undertaken by this working group. Overall, the group developed over 200 maturity statements (Supplementary material 1). Further, it is a key factor that differentiates the CTME MM from other informatics maturity models, for example, the Social and Environmental Determinants of Health MM [16] or the Research IT [15] model that utilized smaller expert development with surveys, or the RIOSM model that was based on extensive interviews across sites.

An unexpected finding of our assessments was the wide range of maturity across the national CTSA hubs. While most CTSAs fall in the mid-range, some institutions rated all axes as 4 or 5, while some others rated all axes a 1 or 2. Across the CTSA consortium, some CTME axes are less mature than others. None of the participating institutions reported level 5 for four of the CTME axes (regulatory and audit management, financial management, subject identification and recruitment, reporting analytics & dashboard). This highlights the need for modern technologies to consolidate data, accelerate review, and improve time to analysis and insights and sheds light on the inherent challenges in improving maturity for some of these axes. Conversely, the CTSA hubs reported higher levels of maturity for some axes such as study management, investigational product management, and subject management. This could be the result of a confluence of phenomena. First, these areas within the CTME activities tend to be more highly regulated. Second, these activities are the focus of many clinical trials management systems implementations. For example, a web-based research management system that has been in operation at the Medical University of South Carolina, known as SPARC, integrates both research and routine clinical care workflows [27,28]. Third, novel initiatives have emerged including the NIH Collaboratory [29], which aims to “rethink clinical trials,” and PCORnet, [30] a national clinical trials infrastructure to facilitate national-level clinical trials to be faster and more efficient by using randomization in the course of routine care and utilizing data from EHRs.

Although our work describes a multi-axial maturity framework to measure organizational capability with respect to the CTME, there are limitations. The recruitment of working group members was opportunistic. Also, the work focused on the CTSA consortium and AMIA, which represent only a portion of the clinical trials activity across the nation. Non-academic and community healthcare organizations were not represented. The results are therefore potentially biased. Further, we acknowledge that not all clinical trial stakeholders, such as the clinical trials regulatory staff and leadership, and those with experience in investigational product management, were directly involved in model development. However, the informatics experts involved in model development work closely on a daily basis with all of these groups. Even so, the maturity statements might not accurately reflect all viewpoints of those involved in clinical trials outside of the informatics community at CTSAs. Although our assessments suggest reasonable real-world applicability, future work must evaluate the generalizability and utility of CTME maturity model beyond the national CTSA hubs.

## Conclusion

The CTME maturity model gives a framework to research organizations to examine their current maturity level with respect to 11 axes relevant to clinical trials and identify areas for future development. It must be borne in mind that this is not a one-time process. Keeping in mind the evolving nature of clinical trials and the complexity of this ecosystem, organizations will need to assess their capabilities on a regular basis and take action as necessary. Future work must determine the optimal frequency for institutional reassessment, and the utility of the maturity model for related activities, such as strategic planning.

## Supporting information

Sehgal et al. supplementary material 1 Sehgal et al. supplementary material

Sehgal et al. supplementary material 2 Sehgal et al. supplementary material

## Supplementary material

For supplementary material accompanying this paper visit https://doi.org/10.1017/cts.2024.1168
