# Supplementary material for: A maturity model for Clinical Trials Management Ecosystem
Source: J Clin Transl Sci. 2025 Jan 22;9(1):e28. doi: 10.1017/cts.2024.1168 (PMC11883580; doi:10.1017/cts.2024.1168)
Supplement: Sehgal et al. supplementary material 1 — Sehgal et al. supplementary material [file S2059866124011683sup001.pdf]

Supplementary Material 1
Clinical Trials Management Ecosystem (CTME) Maturity Model
Levels 1-5 Candidate Statements

Table of contents (linked)

Primary axes/ categories

Generic description of maturity levels

- Level 5 - Optimizing (Efficient)
- Level 4 - Quantitatively Managed (Capable)
- Level 3 - Defined (Aspiring)
- Level 2 - Localized (Developing)
- Level 1 - Initial (Ad hoc)

Definitions and Guides

Maturity level statements

- 1. Study management (Protocol development, routing, and review, CRF development, contract management)
- 2. Regulatory and audit management (Electronic study binder, IRB submissions and approvals)
- 3. Financial management (Charge capture, budgeting, and pricing)
- 4. Investigational product management (Medical equipment, drugs, biomaterial management, research pharmacy, etc.)
- 5. Subject identification and recruitment (Cohort identification, screening, eligibility, consenting, etc.)
- 6. Subject management (Patient study calendar, quality control- adverse event, and cohort management-patient ranking, screening, eligibility, etc.)
- 7. Data Capture and CRF (21 CFR Part 11 compliance, standards-based data management and ontologies, data sharing and governance)
- 8. Reporting, analytics, and dashboard (Report generation, visualization, and dashboards)
- 9. System integration and interfaces (eIRB, Barcode system, enterprise resource planning)
- 10. Staff training and personnel management (Training resources for staff)
- 11. Organizational maturity and culture (CRTO or CRO)

Primary axes/ categories

**Categories**
Study management
Regulatory and audit management
Financial management
Investigational product management
Subject identification and recruitment
Subject management
Data
Reporting, analytics, and dashboard
System integration and interfaces
Staff training and personnel management
Organizational maturity and culture

Generic description of maturity levels

Level 5 - Optimizing (Efficient)

This is the top level - as good as it is going to get. It is the idealized state. It is a characteristic of processes and systems at this level that the focus is on continually improving process performance through both incremental and innovative technological changes/improvements. At maturity level 5, processes are concerned with addressing statistical common causes of process variation and changing the process (for example, to shift the mean of the process performance) to improve process performance.

Level 4 - Quantitatively Managed (Capable)

It is characteristic of processes and systems at this level that using process metrics, effective achievement of the process objectives can be evidenced across a range of operational conditions. The suitability of the process in multiple environments has been tested and the process refined and adapted. Process users have experienced the process in multiple and varied conditions and are able to demonstrate competence. The process maturity enables adaptations to particular projects without measurable losses of quality or deviations from specifications. Process Capability is established from this level. (Example - surgeon performing an operation hundreds of times with levels of negative outcome approaching zero).

Level 3 - Defined (Aspiring)

It is characteristic of processes and systems at this level that there are sets of defined and documented standard processes established and subject to some degree of improvement over time. These standard processes are in place. The processes may not have been systematically or repeatedly used - sufficient for the users to become competent or the process to be validated in a range of situations. This could be considered a developmental stage - with use in a wider range of conditions and user competence development the process can develop to the next level of maturity.

Level 2 - Localized (Developing)

It is characteristic of this level of maturity that some processes are repeatable, possibly with consistent results. Process discipline is unlikely to be rigorous, but where it exists, it may help to ensure that existing processes are maintained during times of stress.

Level 1 - Initial (Ad hoc)

Lowest possible level. This is where you are if you are just starting out. It is characteristic of processes at this level that they are (typically) undocumented, and in a state of dynamic change, tending to be driven in an ad hoc, uncontrolled, and reactive manner by users or events. This provides a chaotic or unstable environment for the processes.

Definitions and Guides

- This assessment may be applied to organizations of various sizes or units within a larger organization. The default definition of “institution” or “organization” would be an academic medical center (AMC) that attempts to function as a cohesive entity regarding clinical research, even if that contains multiple corporate entities.
- In selecting levels, choose the best fit, acknowledging that an organization may satisfy most but not all of the statements regarding the best match level.

Maturity level statements

1. *Study management (Protocol development, routing, and review, CRF development, contract management)*

I. Level 5: Optimizing (Efficient)
- Standardization
  - Electronic systems for study management processes are all implemented and centralized across the institution. (clinical trial protocols, contracts, case report forms, institutional review board (IRB) submissions etc.)
- Complexity/ Integration
  - Policy, processes, and workflow have been optimized and are centralized, standardized, and integrate seamlessly
  - Processes either linked or facilitated by use of the clinical trial management system (CTMS)
- Monitoring
  - Consistent efforts to identify and predict potential issues and for further optimization

II. Level 4: Quantitatively Managed (Capable)
- Standardization
  - Centralized, standardized processes in place across institution.
- Complexity/Integration
  - Electronic systems for study management processes (clinical trial protocols, contracts, case report forms, IRB submissions etc.) are implemented/integrated with minimal need for manual communication between systems (e.g., external IRB web application does not "read" protocols automatically, or EHR does not automatically flag patients on study).
  - Processes either linked or facilitated by use of the CTMS. Information systems relevant to processes are integrated sufficiently to support a single "source" of information but not all systems are integrated so some multiple entry is required.
- Monitoring
  - Consistent efforts to identify and predict potential issues; working towards full integration.

III. Level 3: Defined (Aspiring)
- Standardization
  - Institutional CTMS installed and used for some but not for all studies.
  - Policies are in place, particularly for all study management processes that impact human safety and research results and

policies or standard operating procedure (SOP)s cover all institutional clinical research, not just clinical trials

- Complexity/Integration
  - Major systems like CTMS and IRB are connected, but not all CTM systems. Electronic study management systems exist for most processes, increasing potential for the full electronic integration.
  - Clear path to integration of processes (clinical trial protocols, contracts, case report forms, IRB submissions etc.) .
- Monitoring
  - Well-defined, well-documented, standardized and monitored workflow.

IV. Level 2: Localized (Developing)

- Standardization: Any of the 3 descriptions
  - Localized standardization: Electronic study management systems for many processes at the department or center level (e.g., within a Cancer Center), but little institutional standardization and not necessarily electronically integrated.
  - Emerging, informal global standardization: Defined, informally documented, but not standardized and monitored workflow.
  - Inconsistent: Different systems and processes at different levels of maturity are used across groups/institutes/centers/departments in the institution.
- Complexity/Integration
  - Study lifecycle requires interacting with multiple systems and documents. No integration of CTM systems.
- Monitoring
  - Standardized study milestones, but only informal SOPs that lack specificity.

V. Level 1: Initial (Ad hoc)

- Standardization
  - Ad-hoc, manual processes (clinical trial protocols, contracts, case report forms, IRB submissions etc.) carried out by independent systems
  - Lack of a well-defined, well-documented, controlled, and standardized mechanism
  - Lack of SOPs and standardized study milestones
- Complexity/ Integration
  - No electronic study management systems for these processes
- Monitoring
  - No established framework for measuring and monitoring performance

2. *Regulatory and audit management (Electronic study binder, IRB submissions and approvals)*

I. Level 5: Optimizing (Efficient)
- Standardization
  - Electronic systems are centralized for regulatory and audit management processes.
  - Outcomes of audits such as corrective and preventive action plans are tracked automatically.
- Complexity/ Integration
  - Well-integrated systems that provide interactive and actionable information that can be easily accessed by the main institutional Clinical research and trials office (CRTO)
  - Systems are 21 CFR Part 11 compliant and are used as appropriate enterprise wide
- Monitoring
  - Data from CTMS is used to generate audit reports that are available for review, decision support, and follow-up
  - Process is automatically generated from all studies for Continuous Quality Improvement (CQI) and to support other regulatory requirements
  - Ongoing and systematic CQI

II. Level 4: Quantitatively Managed (Capable)
- Standardization
  - Use of metrics for process management and control are standardized across the institution.
  - Use of industry standard documentation classification
  - Outcomes of audits such as corrective and preventive action plans are documented and completion is tracked
- Complexity/ Integration
  - Some components of audit reports are automatically generated using data from CTMS but may still require double documentation.
  - Automatic labeling in Electronic trial master file (eTMF)
  - Institution has an electronic study binder system, but its use is not mandatory.
- Monitoring
  - Consistent success activating studies on time and reaching study milestones.
  - Study milestones are monitored automatically, but intervention on specific studies is often ad hoc.

III. Level 3: Defined (Aspiring)
- Standardization

- Some electronic systems exist but are not centralized for regulatory and audit management processes.
- Institution-wide standardized audit policies.
- Standardized SOPs or other Quality Management System (QMS) documentation is in place requiring all clinical studies to set up and maintain a regulatory binder (paper or electronic).

- Complexity/ Integration
  - Regulatory binder procedures require all ICH E6 essential documents to be included and managed as controlled documents - i.e.,the regulatory binder procedures are complete {over Good Clinical Practice (GCP)}, apply to all studies regardless of funding source or institutional unit/school/department managing the study.
- Monitoring
  - Established electronic framework for measuring and monitoring performance.

IV. Level 2: Localized (Developing)

- Standardization
  - Inconsistent use of regulatory binders, not standardized across institution.
  - Some key processes are standardized.
- Complexity/ Integration:
  - Event reporting systems such as adverse event (AE), protocol deviation, etc.have been implemented, but response to AEs is still largely manual.
  - Not all ICH E6 essential documents are included in regulatory binders or managed as controlled documents.
- Monitoring:
  - Performance metrics are measured for key areas only

V. Level 1: Initial (Ad hoc)

- Standardization
  - Processes lack a well-defined, well-documented, controlled, and standardized mechanism
- Complexity/ Integration
  - Regulatory and audit paper forms processes.
  - Ad-hoc processes by regulatory and audit management staff and study teams
  - Difficulty activating studies on time and hitting timeline milestones.
- Monitoring
  - No established framework for measuring and monitoring performance or milestones.

3. Financial management (Charge capture, budgeting, and pricing)

I. Level 5: Optimizing (Efficient)
- Standardization:
  - Governance, policy, processes, and workflow for budgeting and pricing have been optimized and standardized into research charge master.
- Complexity/ Integration:
  - CTMS is tightly integrated with electronic health record (EHR)s; automatic, protocol-driven assignment of research vs clinical charges.
  - Integrated reporting that automatically draws data from all relevant systems.
  - Charge-capture automated in relevant systems
- Monitoring
  - Consistent efforts to identify and predict potential issues and for further optimization.
  - Mature budgeting, financial tracking, and forecasting.

II. Level 4: Quantitatively Managed (Capable)
- Standardization:
  - The institutional procedures for pricing, budgeting, financial reporting and financial management of clinical studies specify how the processes are controlled and how process adherence is documented.
  - Metrics needed for process management and control are standardized across the institution.
- Complexity/ Integration:
  - Full CTMS, EHR, and financial integration for high-priority charge capture, with a defined process for completing in other areas.
  - Budget charge capture compliance
  - The processes are auditable (done most efficiently and effectively using system data or other artifacts generated through the process itself).
- Monitoring:
  - Institutional study financial management procedures have mechanisms in place to catch severe under-budgeting studies and conflicts of commitment prior to submission of funding proposals.
  - Centralized financial dashboards for clinical trials portfolio monitoring

III. Level 3: Defined (Aspiring)
- Standardization:

  - Standardized procedures for pricing, budgeting, financial reporting and financial management are in place and apply to all clinical studies at the institution.
  - Centralized charge masters
  - Cost models and prices are institutionally agreed, maintained, and cover all clinical study procedures, services, and supplies.
  - All institutional groups (departments, research labs, institutes and centers, clinical research support units) managing clinical studies have dedicated personnel for pricing, budgeting, financial reporting and management.
- Complexity/ Integration:
  - Institutional procedures cover the full lifecycle of pricing, budgeting, financial reporting and management of clinical studies and apply to all studies regardless of funding source or institutional unit/school/department managing the study.
- Monitoring:
  - The processes are auditable.

IV. Level 2: Localized (Developing)

- Standardization:
  - Many but not all institutional groups (departments, research labs, Institutes and centers, clinical research support units) managing clinical studies have dedicated personnel and procedures for pricing, budgeting, financial reporting and management.
- Complexity/ Integration:
  - Clinical trial study budget pricing lives in an excel document emailed out to teams.
  - Use of shadow financial systems
- Monitoring:
  - Audits are event driven.

V. Level 1: Initial (Ad hoc)

- Standardization:
  - Ad-hoc processes for financial management
  - Manual financial reporting with no single source of truth resulting in conflicting reports.
- Complexity/ Integration:
  - No integration with hospital billing. The business office manually charges insurance or the grant
  - Lack of up-to-date, comprehensive charge master, resulting in inconsistent costing among studies.
  - Slow, manual budget development and pricing based on best guesses for completing protocol procedures

- Monitoring:
  - Monitoring is left to individual units.

4. Investigational product management (Medical equipment, drugs, biomaterial management, research pharmacy, etc.)

I. Level 5: Optimizing (Efficient)
  - Standardization
    - Investigational product use is charged automatically by an integrated system to the grant.
  - Complexity/ Integration
    - The research pharmacy (and management of investigational devices) is managed in a comprehensive and integrated manner, including the inventory of investigational product, though the randomization scheme and subject ID are integrated with the data system collecting protocol data
    - Integrated records linked to barcoded storage labels, system prompts with expiration dates, etc.
    - Able to run double-blinded study with EHR integration and appropriate blinding and unblinding
  - Monitoring
    - Systematic CQI

II. Level 4: Quantitatively Managed (Capable)
  - Standardization
    - Procedures and physical infrastructure for management of investigational products are standardized across the institution.
    - Metrics for process control are standardized across the institution.
    - Organization has implemented a formal controlled document procedure for investigational product management.
  - Complexity/ Integration
    - Investigational Drug Service (IDS) utilizes an institutional platform for managing investigational drug products, including trial status, study team, patient enrollment, IRB expiration, etc.
    - Such a system is integrated with the EHR to charge costs to research studies as needed and for teams to place and track orders.
  - Monitoring
    - Institutional procedures for management of investigational products effectively support the auditing program, identify and lead to resolution of problems.

III. Level 3: Defined (Aspiring)
  - Standardization

    - Institution-wide policies and physical infrastructure for managing investigational products exist, but the implementation of those policies may be inconsistent among research groups
    - These procedures may or may not be managed as controlled documents.
- Complexity/ Integration
    - Institutional procedures in place for an organization that serves as a coordinating center for a multicenter study to ensure that clinical studies specify and control management of investigational products.
    - Institutional procedures for management of investigational product management specify the documentation to be generated by the procedures to enable process control.
- Monitoring
    - Procedures for management of investigational products are auditable.

IV. Level 2: Localized (Developing)

- Standardization
    - Many but not all institutional groups managing clinical studies have procedures and physical infrastructure for managing investigational products when the institution is a site in a multicenter study.
    - Single site studies may or may not have formalized procedures
    - Institutional procedures for an organization that serves as a coordinating center for a multicenter study to ensure that clinical studies specify and control management of investigational products are in place for many but not all institutional groups.
- Complexity/ Integration
    - Effective manual systems in place, but ad hoc integration with other functions, such as billing.
    - Organization emphasized acquisition of information systems versus development of processes.
    - Systems may exist but are not integrated with other systems and use is not standardized and may be redundant.

V. Level 1: Initial (Ad hoc)

- Standardization
    - Ad-hoc, paper-based tracking of stored equipments and drugs, checklist for management
    - Processes lack a well-defined, well-documented, controlled, and standardized mechanism
- Complexity/ Integration

- No electronic system in place for managing investigational products
- No integration with hospital billing system

- Monitoring
    - No established framework for measuring and monitoring performance
    - Crisis responses necessary to address issues with clinical trials.

5. Subject identification and recruitment (Cohort identification, screening, eligibility, consenting, etc.)

I. Level 5: Optimizing (Efficient)
- Standardization
  - Single source of truth for each data type for cohort identification and recruiting
- Complexity/ Integration
  - Integrated system for managing multiple recruitment strategies across varied populations.
  - Integrated consent management, including prior consents related to future studies.
  - Routine process leverage centralized tools interfaced to the EHR enables researchers to identify likely subjects and (multiple) coordinators to work together to screen and recruit them.
  - Clinical research/trials management informs and improves EHR data collection {e.g. Race, ethnicity, Social Determinants of Health (SDOH), etc}
  - Integration of external data sources (e.g., SDOH, community, etc.) for identification of cohorts.
  - Integrated tools for assessing JEDI (Justice, Equity, Diversity, Inclusion) factors in identification/recruitment protocols.
  - Processes to avoid competing or conflicting recruitment activities among studies.
- Monitoring
  - Real-time monitoring of recruitment by study with proactive intervention for low-recruiting studies.

II. Level 4: Quantitatively Managed (Capable)
- Standardization
  - Organization has implemented uniform practices for identification and recruitment of subjects.
  - Centralized mechanisms for managing “do not call” patient preferences regarding recruitment.
  - Centralized systems for subject screening and recruitment.
- Complexity/ Integration
  - Standard examples of integrating identification with recruitment activities.
  - Tools for cohort identification are installed and functional
- Monitoring

- Recruitment monitored at the institutional level.

III. Level 3: Defined (Aspiring)
- Standardization
  - Effective electronic phenotyping accessible to research teams. Ad hoc communication with providers.
  - Processes defined for using clinical data, clinical registries, research/ volunteer, and other registries for identification and recruitment.
  - Policies exist for use of social and other media, such as Facebook, Twitter, Instagram
  - Policies defined for contacting potential research subjects but implementation will vary
- Complexity/ Integration
  - Possible competition for recruitment between study teams.
  - Self service tools available for cohort feasibility analysis.
- Monitoring
  - Periodic, less than annual, monitoring of recruitment.

IV. Level 2: Localized (Developing)
- Standardization
  - Institutional procedures exist for cohort identification, but are not universally applied.
- Complexity.
  - Recruitment coordination at the department or center level, but not institution-wide.
- Monitoring
  - Recruitment evaluation only performed at annual study reports.

V. Level 1: Initial (Ad hoc)
- Standardization
  - Patient referrals, screening and eligibility is carried out using tools developed by the investigators only.
- Complexity/ Integration
  - Inconsistent communication to care providers about research opportunities.
  - Patients may be contacted by multiple simultaneous studies.
- Monitoring
  - No monitoring procedures or infrastructure beyond individual studies.

6. *Subject management (Patient study calendar, quality control- adverse event, and cohort management-patient ranking, screening, eligibility, etc.)*

I. Level 5: Optimizing (Efficient)
- Standardization
  - CTMS is the single source of truth for subject management.
  - Tools and process for management CR across the lifespan (children, teens, adults, elderly, diminished capacity, etc.)
  - Centralized AE reporting, management and adjudication systems
- Complexity/ Integration
  - Interface between CTMS and EHR enables coordination of clinical and research activities at the encounter and procedure level.
  - Integrated biospecimens management including a labeling system that is integrated in the data management system, and a centralized storage system to facilitate easy retrieval
  - Seamless integration with the IRB system for reporting AEs.
  - Tools and processes to enable efficient communication with research subjects.
- Monitoring
  - Automated detection and monitoring of AEs through EHR data

II. Level 4: Quantitatively Managed (Capable)
- Standardization
  - Institutional CTMS tracks subjects on all clinical trials.
  - Institution has standardized on a central patient study calendar system.
- Complexity/ Integration
  - The institution utilizes a comprehensive patient study calendar management, integrated to other CTM systems.
  - The system allows for task lists and reporting for all fields. It is also utilized for Biospecimen collection and is integrated with the EHR at the level of patient and protocol information.
  - The clinical trial AE system is integrated incompletely with relevant systems.
- Monitoring
  - Capability to collect data on trial activity to monitor research metrics that drive decision making and resource management.

III. Level 3: Defined (Aspiring)
- Standardization

    - Institutional CTMS tracks subjects on interventional clinical trials., but not all studies.
    - Use of the patient study calendar varies by study.
- Complexity/ Integration
    - AEs are managed via institutional systems but monitoring is ad hoc and event driven.
    - The AE system is not currently integrated with the IRB.
    - CTMS sends research status to EHR.
- Monitoring
    - Institutional monitoring of subjects on study, but not of protocol progress.

IV. Level 2: Localized (Developing)

- Standardization
    - CTMS exists but not all features are enabled and it is not yet used for all studies to track enrollment.
    - No institutional patient study calendar, but some groups may have a study calendar system.
- Complexity
    - System for AE reporting exists, but the data entry is not currently required by the institution so reporting is unreliable globally.
    - Flagging of research patients in EHR must be entered manually.
- Monitoring
    - Annual study reporting.

V. Level 1: Initial (Ad hoc)

- Standardization
    - Ad hoc systems for AE developed by individual investigators, resulting in delayed or missed AE reporting
- Complexity/ Integration
    - Paper based calendaring approaches
    - Manual integration by research coordinators
    - Poor communication to care providers about patients on studies
    - No tracking in EHR of patients in research studies
- Monitoring
    - Study monitoring occurs in individual units and is driven by study sponsor requirements.

7. Data Capture and CRF (21 CFR Part 11 compliance, standards-based data management and ontologies, data sharing and governance)

I. Level 5: Optimizing (Efficient)
- Standardization
  - Data models and standards are constantly updated and refined to support operational needs and align with national and international standards.
- Complexity/ Integration
  - Integrated calendar-driven system that prompts for inputs and manages the data
  - Data for eCRFs are automatically populated from EHR data across organizations using appropriate standards {e.g., Use of eSource (SMART on Fast Healthcare Interoperability Resources (FHIR)} type interfaces)
  - Data from patient reported outcomes (PRO)s, wearable devices, etc. can be integrated into the master file
  - Data can be exported after database lock in a format that is compliant with current FDA requirements and is easily exported into analytical software.
  - Centralized data governance and coordination infrastructure in place
  - Integration of external data sources {e.g., SDOH, exposure, learning health system (LHS)}
- Monitoring
  - Routine institutional standards for data quality and methods for assessing and responding to data quality issues
  - Financial monitoring and forecasting is used to predict future clinical research resource needs
  - Monitoring of study data management plan used to improve institutional data management policies

II. Level 4: Quantitatively Managed (Capable)
- Standardization
  - Institutional procedures for management of clinical study data are standardized across the institution in alignment with national and international data standards, such as, National Institutes of Health (NIH), Common Data Elements (CDEs), Clinical Data Interchange Standards Consortium (CDISC), Observational Medical Outcomes Partnership (OMOP)

  - Institution mandates FAIR (Findable, Accessible, Interoperable, and Reusable) principles for data sharing
- Complexity/ Integration
  - Centralized institutional resources exist for management of CR data.
  - Common eCRF (electronic case report form) data elements are defined across the institution.
  - eCRF import of EHR data is available, but not universally used.
- Monitoring
  - Institutional procedures for management and sharing of clinical study data effectively support the auditing program, identify and lead to resolution of problems.
  - Institutional policies for monitoring compliance with data management plans.

III. Level 3: Defined (Aspiring)

- Standardization
  - Institution-wide policies for managing data exist, but the implementation of those policies may be inconsistent among research groups
  - These procedures may or may not be managed as controlled documents.
- Complexity/ Integration
  - Processes defined for integration of CRFs and documentation templates.
  - Institutional procedures in place for an organization that serves as a coordinating center for a multicenter study to ensure that clinical studies specify and control management of data.
  - Institutional procedures for management of data specify the documentation to be generated by the procedures to enable process control.
  - 21 CFR Part 11 compliant electronic data capture (EDC) is available as an institutional resource, but is not universally used.
- Monitoring
  - Institutional procedures for development of a data management plan for clinical studies specifying the documentation to be generated by the procedures to enable auditability and traceability of all changes to data.

IV. Level 2: Localized (Developing)

- Standardization

  - Many but not all institutional groups managing clinical studies have procedures for managing data when the institution is a site in a multicenter study.
  - These procedures may or may not be exhaustive and may not be managed as study-level controlled documents.
  - Single site studies may or may not have formalized data management procedures
  - Institutional procedures for an organization that serves as a coordinating center for a multicenter study to ensure that clinical studies specify and control management of processes for managing study data, such as creation of data management plans, access control to study data, selection of data sources, etc. are in place for many but not all institutional groups.
- Complexity/ Integration
  - Systems may exist but are not integrated with other systems and use is not standardized and may be redundant.
  - Currently in the process of implementing a 21 CFR Part 11 compliant EDC. The system is integrated and being piloted, but not fully compliant. Most other electronic data capture is through industry sponsor systems.
- Monitoring
  - Event driven data management audits.

V. Level 1: Initial (Ad hoc)

- Standardization
  - No standardized solution in place
  - Ad-hoc process by service providers, study teams and requestors. Paper based data collection for clinical trials
- Complexity/ Integration
  - No integration between source systems. Data from other sources (such as the EHR) must be entered manually by data managers into multiple systems.
- Monitoring
  - Manual processes for locking study data and generating regulatory (e.g., FDA) reports.
  - Lack of data quality processes

8. Reporting, analytics, and dashboard (Report generation, visualization, and dashboards)

I. Level 5: Optimizing (Efficient)
- Standardization
  - Organizational standardized and automated processes to review reports and modify behaviors as needed.
  - Self-serve reporting capability using standardized fields
  - Single source of truth for reports. Report content is reliable and repeatable
  - Standardized process for reviewing existing reports and reporting tools and proactive development/retirement of reports.
- Complexity/ Integration
  - Reporting dashboards are available for all departments via tools like PowerBi or tableau, dynamically linked to electronic data warehouse (EDW) tables
  - Both canned and ad-hoc report generation is centrally managed, cataloged and automated where applicable for both scalability and correctness.
  - Interfaces for seamless generation of multi-site reports and interaction with external monitors.
- Monitoring
  - Reporting supports predictive analytics on the study and institutional basis.
  - Reporting drives a continuous improvement cycle.
  - Mature metadata management

II. Level 4: Quantitatively Managed (Capable)
- Standardization
  - Institutional procedures for management of planned and post-hoc analyses are standardized across the institution
  - These procedures are managed as controlled documents.
- Complexity/ Integration
  - Centralized, institutional resources exist for reporting and analytics
  - Highly effective business analytics beginning to impact clinical services esp. with respect to quality improvement.
- Monitoring
  - Institutional procedures for reporting and analytics effectively support the auditing program, identify and lead to resolution of problems.
  - Dashboards and reports are designed to produce actionable information.

III. Level 3: Defined (Aspiring)

- Standardization
  - Institution-wide policies for planned and post hoc analyses exist, but the implementation of those policies may be inconsistent among research groups
  - These procedures may or may not be managed as controlled documents.
- Complexity/ Integration
  - Institutional procedures for planned and post-hoc analyses specify the documentation to be generated by the procedures to enable process control.
- Monitoring
  - Institutional procedures for design and management of planned and post-hoc analyses specify the documentation to be generated by the procedures to enable auditability and traceability of all operations performed on data such as exclusion, censoring, imputation, classification, etc.

IV. Level 2: Localized (Developing)

- Standardization
  - Many but not all institutional groups managing clinical studies have procedures for assuring that clinical studies specify and control processes for designing, programming, validating, and reporting planned study analyses and for handling and documenting unplanned analyses.
- Complexity/ Integration
  - Existing institute-specific procedures may or may not be exhaustive and may not be managed as controlled documents.
- Monitoring
  - Monitoring of reports may be done by individual groups or departments, but not institutionally coordinated.
  - Reports and dashboards provide information but are difficult to convert into process improvement projects.

V. Level 1: Initial (Ad hoc)

- Standardization
  - Lack of institution wide reports.
  - Ad-hoc operations for reporting, analytics and visualization
  - Lack of pathways for access to standardized data for reporting
- Complexity/ Integration
  - No dashboards available. Simple tables or spreadsheets.

    - Unreliable, inconsistent and unrepeatable outcomes with no validation
- Monitoring
    - No forecasting or predictive reporting.
    - Monitoring data captured by different groups in inconsistent formats

9. System integration and interfaces (eIRB, Barcode system, enterprise resource planning)

I. Level 5: Optimizing (Efficient)
- Standardization
  - All systems are tightly integrated but loosely coupled via a Service Oriented Architecture (SOA).
  - Standards-based interfaces. IHE (Integrating the healthcare enterprise), FHIR and other electronic interfaces.
  - Institutional standards and processes for interface change management.
- Complexity/ Integration
  - Well-documented integration and interfaces between systems with proper meta-data and contextual information to ensure meaningful integration and interface between systems.
  - Seamless integration among (EHR, CTMS, IRB EDC) across organization and to external organizations.
  - Organization level Service-Level Agreement (SLA) / Uptime
  - Single sign-on for all CTME related systems.
- Monitoring
  - All systems have access to the most current relevant data (e.g. real-time or near real-time interfaces, or event-driven data distribution).

II. Level 4: Quantitatively Managed (Capable)
- Standardization
  - Institutional policies for process and system integration and interfaces between systems are standardized across the institution.
  - Institutional standards for documentation of interfaces.
- Complexity/ Integration
  - Integration of major systems (e.g., EHR, CTMS, IRB) within the organization.
  - Institutional policies for interface SLAs exist, but are not uniformly implemented.
- Monitoring
  - Routine real-time monitoring of integration status.

III. Level 3: Defined (Aspiring)
- Standardization

    - Institutional policies for process and system integration and interfaces exist but implementation is inconsistent among research groups.
- Complexity/ Integration
    - Initial work on integration of major systems (e.g. EHR, CTMS, IRB)
    - Institutional focus on integration of clinical care systems, with relatively little institutional focus on integration between clinical and research systems.
- Monitoring
    - Routine periodic monitoring of system integration (e.g. daily/weekly)

IV. Level 2: Localized (Developing)

- Standardization
    - Many but not all institutional groups/ departments have policies in place for integration and interfaces of systems and processes.
- Complexity/ Integration
    - Systems may exist but are minimally integrated with other systems and use is not standardized and may be redundant.
    - Lack of integration results in duplicative processes and inconsistent data
- Monitoring
    - Event driven monitoring in response to reported failures.

V. Level 1: Initial (Ad hoc)

- Standardization
    - Data entry into multiple systems, with manual reconciliation
- Complexity/ Integration
    - No integration among various systems, except ad-hoc study-specific data integrations
- Monitoring
    - Little if any monitoring of system integrations.

10. Staff training and personnel management (Training resources for staff)

I. Level 5: Optimizing (Efficient)
- Standardization
  - Organization has standardized, comprehensive policies and procedures for CR workforce development, management, and career progression.
  - Organizational policies ensure training on appropriate systems at onboarding and periodically.
- Complexity/ Integration
  - Web-based, scalable trainings (GCP, HSP, or specific study trainings) available
  - Centralized tracking of relevant training with automated reminders
    a. Tracking system integrated with other systems for enforcement (e.g., expired IRB training will automatically revoke staff access to relevant systems such as IRB, patient scheduling system, etc.)
  - Training content constantly updated, refined and optimized
- Monitoring
  - Training outcome, effectiveness, and impact are constantly monitored.
  - AE and trial performance monitoring informs development of training modules.

II. Level 4: Quantitatively Managed (Capable)
- Standardization
  - Institutional policies and procedures for CR workforce development and management are standardized across the institution.
  - These procedures are managed as controlled documents.
  - Institutionally standardized roles and responsibilities
  - Standardized onboarding training based on roles, responsibilities, and job functions
- Complexity/ Integration
  - Centralized, institutional resources exist for staff training.
  - Web-based systems to track and manage training requests.
- Monitoring
  - Institutional procedures for staff training and personnel management effectively support the auditing program, identify and lead to resolution of problems.

III. Level: 3: Defined (Aspiring)

- Standardization
  - Institutional policies and procedures for CR workforce training and management exist but the implementation of those policies may be inconsistent among research groups.
  - These procedures may or may not be managed as controlled documents.
  - Major roles and responsibilities are standardized
  - Onboarding training for basic and common job functions, but ad hoc for others
- Complexity/ Integration
  - Institutional procedures for CR workforce training and management specify the documentation to be generated by the procedures to enable process control.
  - Some groups have adopted centralized, online training resources.
- Monitoring
  - Institutional procedures for CR workforce training and management specify the documentation to be generated by the procedures to enable auditability and traceability of personnel working on each study.

IV. Level 2: Localized (Developing)

- Standardization
  - Many but not all institutional groups/ departments have policies in place for CR workforce training and management.
  - Some groups/ departments have defined roles and responsibilities.
  - Organization considering harmonization of roles and responsibilities across the institution.
  - Much training is peer to peer and reactive rather than standardized and proactive.
- Complexity/ Integration
  - Training for staff exists but timing and needs are not always well matched.
- Monitoring
  - Periodic (e.g. annual) monitoring means that some CR staff may begin CR activities before full training has been completed.

V. Level 1: Initial (Ad hoc)

- Standardization
  - Required training limited to Good Clinical Practice (GCP) and human subjects protections (HSP) with little standardization of training provider.

    - No standardization of CR roles, positions, responsibilities, or advanced training.
- Complexity/ integration
    - No centralized tracking of training
    - No training for local systems policies or procedures
- Monitoring
    - Little if any monitoring of staff training.

11. Organizational maturity and culture (CRTO or CRO)

I. Level 5: Optimizing (Efficient)
- Standardization
  - Top-level leadership commitment to be a leading clinical research organization
  - Centralized research organization is well thought out and structured to accomplish a well-defined set of missions and goals
    a. This structure is continuously reviewed, refined and adjusted to address and support constant changes of the environment.
  - CR governance incorporates input from all stakeholder groups.
- Complexity/ Integration
  - Organizational culture is built and ingrained to support system thinking, collaboration and service-oriented mentality of the staff
  - Organizational commitment to participation in relevant national organizations/meetings.
  - All levels of the clinical organization actively promote that clinical research is an integral part of the clinical mission (including commitment of money and staff time).
  - Research leadership promotes the importance of clinical research, and promotes clinical research activities to support a complete translational spectrum.
  - Proactive mechanism exists for feasibility assessment that minimizes the chance of resource competition
  - Disease specific centers, e.g. cancer centers, if present, are seamlessly integrated across all CR functions.
- Monitoring
  - Each organization's effectiveness is constantly monitored and measured to optimize its function.
  - Proactive procedures in place to identify, evaluate, and adopt best practices in clinical research.

II. Level 4: Quantitatively Managed (Capable)
- Standardization
  - Top-level leadership across the organization is committed to achieving a set of clinical research missions and goals.
  - Centralized CR governance group includes some, but not all, stakeholders.
- Complexity/ Integration

- Organizational commitment (money, staff) to promote clinical research.
- Institutional CTO or equivalent oversees all clinical research.
- Some departments have mechanisms for minimizing resource competition between studies but no proactive institutional mechanism.
- Disease specific centers, e.g. cancer centers, if present, collaborate with other CR functions.

- Monitoring
  - Centralized institutional resources exist for monitoring organization's effectiveness, identifying and resolving problems.

III. Level 3: Defined (Aspiring)

- Standardization
  - Institutional leadership acknowledges the importance of clinical research, but support throughout the organization is variable.
  - CR governance is assigned to another institutional governance group, no dedicated CR governance group.
- Complexity/ Integration
  - CTO or equivalent exists but may not oversee all clinical research.
  - Integration of clinical research and care occurs at the department/ research group level.
  - Mechanisms exist for responding to and resolving conflicts over resources.
  - Disease specific centers, e.g. cancer centers, if present, are aligned with other CR functions.
- Monitoring
  - Institution periodically monitors CR maturity.

IV. Level 2: Localized (Developing)

- Standardization
  - Some but not all institutional groups/ departments have leadership committed to achieving a set of clinical research missions and goals
  - Clinical organization allows but does not prioritize clinical research
- Complexity/Integration
  - Clinical Trials Office under development.
  - Gaps in organizational structure are recognized and beginning to be addressed but no organizational priority to grow clinical research.
  - Disease specific centers, e.g. cancer centers, if present, are siloed.
- Monitoring

    - Monitoring of organizational maturity may be done by individual groups or departments, but not institutionally coordinated.

V. Level 1: Initial (Ad hoc)

- Standardization
    - Lack of centralization / institutionalization
    - No institutional commitment to clinical research
- Complexity/ Integration
    - Research organizational structure is outdated and/or poorly structured and does not align with workflow, function and responsibilities
    - Gaps in organizational structure resulting in confusion, inconsistency, and redundancy
    - Key and/or important support/services are missing due to poor organizational structure
    - Poor organization culture leads to siloed thinking, mentality and operation
    - Frequent conflict/competition among multiple research groups.
- Monitoring
    - Little if any monitoring of organizational maturity.
