# Supplementary material for: A maturity model for Clinical Trials Management Ecosystem
Source: J Clin Transl Sci. 2025 Jan 22;9(1):e28. doi: 10.1017/cts.2024.1168 (PMC11883580; doi:10.1017/cts.2024.1168)
Supplement: Sehgal et al. supplementary material 2 — Sehgal et al. supplementary material [file S2059866124011683sup002.pdf]

Supplementary Material 2. Summary of CTME Maturity Scores by Institution.
CTME: Clinical Trials Management Ecosystem

			**SITE ID**																						
**Axis**	**Mean**	**S.D.**	**1**	**2**	**3**	**4**	**5**	**6**	**7**	**8**	**9**	**10**	**11**	**12**	**13**	**14**	**15**	**16**	**17**	**18**	**19**	**20**	**21**	**22**	**23**
**Study Management**	**3.09**	**1.12**	2	2	3	3	4	5	4	2	3	4	4	3	4	5	2	2	3	1	3	2	3	2	5
**Regulatory and Audit Management**	**2.73**	**0.88**	1	2	3	3	4	4	2	2	2	3	4	3	3	4	3	2	3	1	3	3	3	2	
**Financial Management**	**2.61**	**0.84**	2	2	2	2	3	4	4	2	3	2	3	3	3	2	4	2	3	1	2	2	3	2	4
**Investigational Product Management**	**3.09**	**1.15**	2	3	3	5	4	5	3	3	4	2	4	4	4	5	3	2	2	1	2	2	3	2	
**Subject Identification and Management**	**2.91**	**0.81**	2	1	4	3	3	4	3	3	3	3	4	4	3	4	3	2	2	2	3	3	3	2	
**Subject Management**	**3.00**	**1.13**	2	2	3	5	4	5	4	2	3	2	4	3	3	5	3	2	3	1	2	2	3	2	4
**Data**	**2.78**	**0.90**	2	1	3	4	3	5	3	2	2	2	3	3	3	2	3	2	3	2	4	3	3	2	4
**Reporting Analytics and Dashboard**	**2.57**	**1.20**	1	1	1	4	3	4	3	1	2	3	4	1	2	4	4	2	3	1	2	3	4	2	4

**System Integration**	**2.96**	**1.07**	1	2	2	4	4	4	3	3	4	3	4	2	3	5	2	2	3	2	3	2	3	2	5
**Staff Training and Personnel Management**	**2.96**	**1.02**	2	2	3	4	4	5	3	3	2	4	4	4	4	3	3	2	3	1	2	2	2	2	4
**Organizational Maturity and Culture**	**2.70**	**1.18**	1	2	1	4	4	4	3	3	3	4	4	3	4	5	2	2	3	1	1	2	2	2	2

Note: Shaded cells indicate no answer by the respondent.
